# The microRNA-200 family: small molecules with novel roles in cancer development, progression and therapy

**DOI:** 10.18632/oncotarget.3052

**Published:** 2015-01-30

**Authors:** Brock Humphries, Chengfeng Yang

**Affiliations:** ^1^ Department of Physiology, Michigan State University, East Lansing, MI 48824, USA; ^2^ Cellular and Molecular Biology Graduate Program, Michigan State University, East Lansing, MI 48824, USA; ^3^ Center for Integrative Toxicology, Michigan State University, East Lansing, MI 48824, USA

**Keywords:** microRNA, miR-200, cancer initiation, cancer metastasis, cancer therapeutic target

## Abstract

MicroRNAs (miRNAs) are a large family of small non-coding RNAs that negatively regulate protein-coding gene expression post-transcriptionally via base pairing between the 5′ seed region of a miRNA and the 3′ untranslated region (3′UTR) of a messenger RNA (mRNA). Recent evidence has supported the critical role that miRNAs play in many diseases including cancer. The miR-200 family consisting of 5 members (miR-200a, -200b, -200c, -141, -429) is an emerging miRNA family that has been shown to play crucial roles in cancer initiation and metastasis, and potentially be important for the diagnosis and treatment of cancer. While miR-200s were found to be critically involved in the metastatic colonization to the lungs in mouse mammary xenograft tumor models, a large number of studies demonstrated their strong suppressive effects on cell transformation, cancer cell proliferation, migration, invasion, tumor growth and metastasis. This review aims to discuss research findings about the role of the miR-200 family in cancer initiation, each step of cancer metastatic cascade, cancer diagnosis and treatment. A comprehensive summary of currently validated miR-200 targets is also presented. It is concluded that miR-200 family may serve as novel targets for the therapy of multiple types of cancer.

## INTRODUCTION

### The miRNAs

The first microRNA (miRNA) (lin-4) was discovered in 1993 [1, 2], however the term “microRNA” wasn't introduced until 2001 [3–5]. Within the next year it was reported for the first time that miRNAs are likely involved in cancer, by demonstrating that miR-15 and -16 were frequently deleted in chronic lymphocytic leukemia (CLL) [6]. Since this discovery a focus has been put on identifying and determining the role of miRNAs involved in cancer development, progression, diagnosis and treatment. Through this effort, our understanding of how microRNAs function and the role they play in cancer has increased tremendously.

MiRNAs are a large family of small non-coding RNA molecules (over 2500 in humans: miRBase.org) that negatively regulate protein-coding gene expression post-transcriptionally. MiRNAs are initially transcribed mono- or polycistronically in the nucleus by RNA polymerase II into primary transcripts (termed primary miRNA, pri-miRNA) ranging from hundreds to thousands of nucleotides long, which are then polyadenylated and capped [7, 8]. These pri-miRNA transcripts are subjected to a microprocessing event carried out by a type III RNase Drosha and its binding partner DiGeorge Syndrome Critical Region 8 (DGCR8) to reduce the size of the transcript to ~70 nucleotides termed precursor- or pre-miRNA [9, 10]. The pre-miRNA is then exported to the cytosol by exportin-5, where it undergoes another processing event performed by another type III RNase Dicer resulting in a ~21–22 nucleotide miRNA duplex [11, 12]. After unwinding, one strand of the duplex is usually degraded, while the other strand, the mature miRNA, is associated with Argonaute and then incorporated into the RNA induced silencing complex (RISC), where miRNAs are then able to regulate the expression of their target genes.

Although it has been shown that miRNAs can interact with other parts of the messenger RNAs (mRNAs) (for example see [13]), miRNAs typically function by base pairing with the 3′ untranslated regions (3′-UTRs) of their target mRNAs through the seed sequences of the miRNAs. The seed sequence of a miRNA is the second to the eighth nucleotide region at the 5′ end of the mature miRNA that generates the specificity of each miRNA to its target mRNAs. The base pairing between a miRNA and its target mRNAs can result in mRNA destabilization and degradation, translational inhibition, or mRNA direct cleavage [14–17]. Down-regulation of mRNAs through miRNA-caused mRNA destabilization and degradation is common, which is usually mediated by the same imperfect miRNA:mRNA base pairing that leads to translational inhibition [18–20]. Conversely, mRNA down-regulation through miRNA-caused mRNA direct cleavage occurs in rare cases, which usually requires more extensive base pairing [16, 17]. While a miRNA's seed sequence is usually the most prominent characteristic that determines the specificity of the miRNA:mRNA interaction, there are examples of miRNAs that have weak seed sequence binding but better overall complementarity which can direct the inhibition of gene expression [15, 21, 22].

MiRNAs are interesting because each can target multiple genes and can also share seed sequences with other miRNAs, therefore theoretically targeting the same genes as other miRNAs. It is for this reason that miRNAs have been thought to regulate upwards of two-thirds of all protein coding genes in humans [23]. Furthermore, miRNAs have been shown to be involved in almost all aspects of cellular functions. Therefore it is probable that miRNAs play critical roles in cancer development and progression. Indeed there is a growing body of evidence suggesting that miRNAs may act as either oncogenes or tumor suppressors, and are involved in the development, progression, and treatment of cancer (for reviews see [24, 25]). These miRNAs that act as either oncogenes or tumor suppressors in a cell are often called oncomirs [26].

### The miRNA-200 family

**Figure 1:**
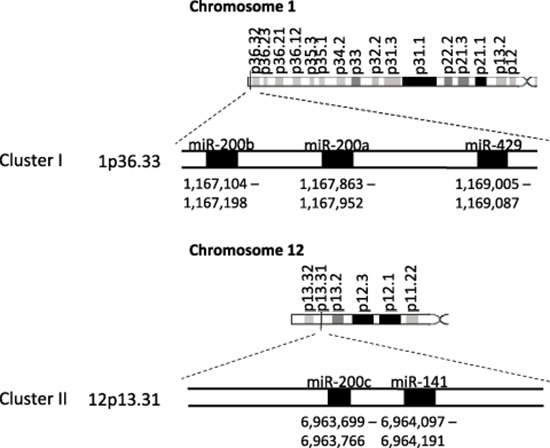
The miR-200 family two clusters are located on two different chromosomes The miR-200 family consists of two clusters: Cluster I (*miR-200b*, -*200a*, and -*429* is located on chromosome 1) and Cluster II (*miR-200c* and -*141* is located on chromosome 12).

**Figure 2:**
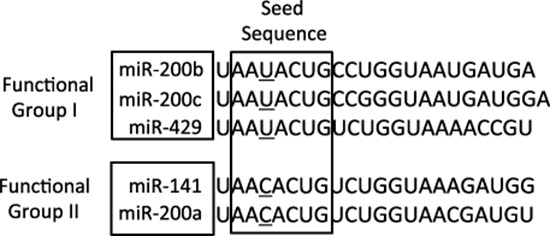
The sequences of the mature miRNA-200 family members The miR-200 family members can also be separated into two functional groups based upon their seed sequences. Functional Group I is composed of miR-200b, -200c, and -429 and Functional Group II consists of miR-141 and -200a. The seed sequences of these two functional groups only differs by one nucleotide: AAUACUG for group I and AACACUG for group II.

The miRNA-200 (miR-200) family consists of five members, which form two clusters located in two different genomic regions. As shown in Figure 1, the Cluster I miR-200s in humans contains *miR-200b*, *-200a*, and *-429* (*miR-200b/200a/429*) located in an intergenic region of chromosome 1, and cluster II miR-200s contains *miR-200c* and *-141* (*miR-200c/141*) located on chromosome 12 [27, 28]. Alternatively, the miR-200 family members can also be divided into two functional groups based upon the similarities of their seed sequences (Figure 2). MiR-200b, -200c, and -429 (Functional Group I) all share the same seed sequence and miR-200a and -141 (Functional Group II) both share the same seed sequence, with the two functional groups only differing in the seed sequence by one nucleotide (AAUACUG for miR-200b/200c/429 and AACACUG for miR-200a/141). The miR-200 family is highly conserved among vertebrate species and highly expressed within epithelial cells.

The expression of the miR-200 family can be regulated through interactions with, and modifications of their promoters. Recent studies suggest that modifications to the promoter regions of each of the miR-200 clusters can cause the loss of the expression of the miR-200 family in cancer. The promoter region of the *miR-200c/-141* cluster has been shown to be hypermethylated [29, 30], whereas the *miR-200b/-200a/-429* cluster has been shown to be silenced primarily through polycomb group-mediated histone modifications [31] in cancer. Alternatively, the promoter regions of the miR-200 family can be bound by the transcription factors zinc finger e-box bind homeobox 1 (ZEB1) and 2 (ZEB2 also known as SIP1), specificity protein 1 (Sp1), and p53. When bound, ZEB1 and ZEB2 can inhibit the transcription of the entire miR-200 family, while Sp1 and p53 binding has been shown to lead to activation of transcription of the *miR-200b/200a/429* [32, 33] and the *miR-200c/141* [33, 34] clusters, respectively. However, Kolesnikoff and colleagues also showed that Sp1-mediated activation of *miR-200b/200a/429* transcription can be disrupted by the expression and interaction of ZEB1/2 with its binding sites within the promoter.

The miR-200 family is among the most widely studied miRNAs in cancer, this review will focus specifically on the role of the miR-200 family in cancer initiation, metastasis, diagnosis and treatment.

## The miR-200 family in cell transformation and tumorigenesis

Tumor initiation is a complex process by which normal cells are transformed into malignant tumor cells, which then produce a tumor. Throughout this process the molecular profile of the cell is changed in such a way as to allow these cells the ability to form a tumor. Recent research has suggested that the miR-200 family plays an important role in inhibiting cell malignant transformation and preventing tumor initiation.

Recent research done in our laboratory has been the first to show an important role of the miR-200 family in inhibiting and preventing cell malignant transformation by a carcinogen exposure [35]. Using immortalized human bronchial epithelial cells (HBECs) that either had normal p53 expression (HBECs) or p53 knocked down (p53^low^HBECs), we were able to show that chronic exposure to a low concentration of arsenic caused epithelial to mesenchymal transition (EMT) and malignant transformation in p53^low^HBECs, but not in p53 intact HBECs. Only the p53^low^HBECs exposed to arsenic formed colonies in soft agar and formed tumors in a subcuteaneous injection of the cells into the nude mice. A miRNA microarray analysis showed that the expression levels of miR-200 family were drastically reduced in arsenic-transformed cells.

To determine whether down-regulation of the miR-200 family plays a role in arsenic-induced cell malignant transformation, we first transiently re-expressed miR-200b or -200c in arsenic-transformed cells and found that re-expression of miR-200b or -200c alone or together restored E-cadherin expression and the epithelial-like cellular morphology, and reduced the formation of colonies in soft agar. Then we generated miR-200b stable expression cells and found that stably re-expressing miR-200b in arsenic-transformed cells abolished their ability to form colonies in soft agar, and tumors in nude mice when injected subcutaneously. Then we overexpressed miR-200b in parental p53^low^HBECs and found that forced expression of miR-200b prevented cellular transformation by chronic low dose arsenic exposure [35]. Together, these findings suggest that loss of miR-200 expression plays a causal role in arsenic-induced cell malignant transformation and tumorigenesis.

A study investigating the mechanism of tobacco carcinogen-induced cell transformation showed that a 4 week treatment of p53 intact HBECs with genotoxic, but not cytotoxic, doses of *N*-Nitroso-*N*-methylurea (NMU) or Benzo(*a*)pyrene diolepoxide (BPDE) caused EMT and cell transformation as evidenced by occurrence of mesenchymal-like cellular morphology and increased soft agar colony formation [36]. Further experiments revealed that expression of miR-200b and -200c were significantly reduced after 4 week carcinogen treatment. Transient or stable expression of these miRNAs in tobacco carcinogen-transformed HBECs restored the epithelial-like cellular morphology and reduced soft agar colony formation [36].

These two studies described above both looked at epigenetic silencing as a possible mechanism for the carcinogen-induced miR-200 expression loss seen in the HBECs. It was determined that the promoter regions of the miR-200 family were indeed highly methylated upon treatment with the carcinogen, and demethylation induced by DNA methyltransferase inhibitors or demethylation chemicals increased the expression of the miR-200 family. Therefore, arsenic or tobacco carcinogens may induce cell transformation by increasing the methylation of the promoter regions of, and subsequently leading to silencing of, the miR-200 family. Together, these studies suggest that loss of miR-200 expression may play an important role in the early stage of carcinogenesis.

## The miR-200 family in cancer metastasis

Cancer metastasis is the result of a multi-step signaling cascade in which cancer cells move away from the primary tumor site to colonize distant organs and form secondary (or metastatic) tumors [37]. Typically, cells that accomplish metastasis have undergone widespread genetic and epigenetic modifications that benefit the survival, growth, invasion and movement of the cell. Metastasis can be broken down into 6 steps [38]: 1) growth and vascularization of the primary tumor, and nearby tissue invasion, 2) detachment of the cancer cells and migration away from the primary tumor, 3) intravasation into the blood stream and lymph nodes, 4) survival and circularization within the blood stream and lymph nodes, 5) attachment to the blood vessel wall and extravasation, and finally 6) colonization of the distant organs and growth of metastatic tumors. Each step symbolizes an important obstacle that the tumor cell must overcome to result in successful metastasis.

Current research on the miR-200 family has shown that the family can affect each step of the metastatic cascade. Therefore, this section will summarize these works in respect to the order of each metastatic step as described above.

### Effect of the miR-200 family on tumor growth, angiogenesis, and nearby tissue invasion

Once a cell undergoes transformation to a malignant cell, the checkpoints that limit its growth are bypassed resulting in uncontrolled growth. This uncontrolled growth leads to the formation of a primary tumor. Not only has the miR-200 family been shown to inhibit cellular malignant transformation, but studies have also shown that they are capable of suppressing tumor growth. For example, it was found that forced expression of miR-200a in meningioma cells [39], or expression of miR-429 in SW260 colorectal carcinoma cells [40], reduced xenograft tumor growth when injected into the flanks of SCID or nude mice, respectively. However, the reported effects of miR-200b on xenograft tumor growth are less consistent as some studies including ours have shown that expression of miR-200b decreased tumor growth [41, 42] while others have shown that it has little or no effect on tumor growth [43, 44]. Therefore more work is needed to determine the role of the miR-200 family in this early step of metastasis.

Once a tumor reaches 1–2 mm^3^ the cells at the center of the tumor are under hypoxic conditions and do not receive enough nutrients to grow [45]. In order to combat this environment a tumor must initiate angiogenesis, which allows new blood vessels to form intricately within the tumor. Recent studies have shown that the miR-200 family can inhibit angiogenesis because the family targets multiple key players in this process.

In this regard, two separate studies have shown that miR-200b directly targets vascular endothelial growth factor A (VEGFA) [46, 47], a ligand that is considered the master determinant for the activation of the angiogenic program. Furthermore, the miR-200 family has also been shown to target the VEGF receptors. For example, in A549 lung cancer cells, Choi and colleagues demonstrated that transient miR-200b expression reduced Flt1 (VEGF receptor 1) and KDR (VEGF receptor 2) protein level, and a luciferase reporter assay confirmed the direct interaction between miR-200b and the 3′UTR of these proteins. Similarly, miR-200c has also been shown to directly target KDR [48]. These findings were further confirmed by Roybal and colleagues showing that stable expression of cluster I miR-200s reduced protein and mRNA levels of the receptor tyrosine kinase Flt1 (VEGFR1) in lung adenocarcinoma 344SQ cells [49]. Additionally, miR-200a and miR-200b have also been shown to directly target the pro-angiogenic ligands interleukin 8 (IL-8) and chemokine (C-X-C motif) ligand 1 (CXCL1) to regulate angiogenesis in ovarian cancer [50]. Taken together, these data suggest that the miR-200 family plays crucial roles in the metastatic cascade by down-regulating important players involved in angiogenesis.

Research in our lab has also shown an important role of miR-200b in inhibiting tumor angiogenesis. Ours and other studies showed that inoculation of arsenic-transformed cells produced invasive and metastatic xenograft tumors in nude mice [51–53], however, the underlying mechanism is not clear. To examine whether arsenic-transformed cells have a pro-angiogenic activity, we tested the effect of conditioned media from these cells on the tube forming ability of human umbilical vein epithelial cells (HUVECs). HUVECs cultured in conditioned media from arsenic-transformed cells formed extensive tubes compared to HUVECs cultured in conditioned media from non-transformed control cells, suggesting a pro-angiogenic capability of arsenic-transformed cells [54]. To study the role of miR-200b in this process, we used conditioned media from arsenic-transformed cells that stably expressed miR-200b. It was found that HUVECs cultured in conditioned media from arsenic-transformed cells stably expressing miR-200b formed significantly less tubes. Furthermore, the immunofluorescence staining of CD31, a marker of blood vessel endothelial cells, on xenograft tissues resulting from the injection of arsenic-transformed cells stably expressing miR-200b showed a drastic reduction compared to CD31 staining on xenograft tumors resulting from the injection of arsenic-transformed vector control cells [54]. Our additional mechanistic studies suggested that this reduction of angiogenesis by miR-200b is likely due to down-regulation of VEGF levels resulting from β-catenin sequestration at the plasma membrane by increased expression of E-cadherin. Together, these findings provide additional evidence supporting that miR-200b is capable of inhibiting tumor angiogenesis.

Tumor nearby tissue invasion involves breakage of the boundaries of tissues where tumors originate from and the entry of cancer cells from the primary tumor into the surrounding stroma. Sossey-Alaoui and colleagues found that WAS protein family member 3 (WAVE3) was critical for the invasive properties of transformed cells [55]. Further analysis revealed that miR-200b directly targets WAVE3 through interaction with its 3′UTR in MDA-MB-231 breast, LNCaP prostate, and HT29 colorectal cancer cells. To study the effects of WAVE3 on cell invasion, a Matrigel-invasion assay showed that cells treated with WAVE3 siRNA or that overexpression of miR-200b reduced the invasive capability, while using an anti-miR-200b oligonucleotide increased the number of invading cells. Expressing a WAVE3 mRNA that is resistant to miR-200b targeting also reversed the inhibitory effects of miR-200b on cell invasion, further suggesting a critical role for miR-200b in the inhibition of WAVE3-dependent cellular invasion.

Another study looked at the effects of miR-200 on targets that regulate the reorganization of the actin cytoskeleton to promote invasiveness [56]. Transient transfection of a miR-200c mimic in MDA-MB-231 breast cancer cells showed a strong inhibitory effect on the invasive capabilities of these cells in Matrigel and when using a real-time cell analyzer. Furthermore, the increase in the level of miR-200c was accompanied by a decrease in stress fiber formation that was not accompanied by a change in RhoA activity, suggesting that miR-200c likely acted downstream of RhoA. Transient transfection of miR-200c reduced both formin homology domain-containing protein 1 (FHOD1) and Mg^2+^/Mn^2+^-dependent protein phosphatase 1F (PPM1F) levels, and inhibition of the miR-200b/c/429 cluster in MCF7 breast cancer cells increased FHOD1 and PPM1F levels. A luciferase reporter assay confirmed that miR-200c directly targets the 3′UTR of these mRNAs in MDA-MB-231, MCF-7, and HEK-293FT cells. Silencing of FHOD1 or PPM1F resulted in decreased invasion in MDA-MB-231 cells using the same assays as before, showing the importance of these targets in invasion.

Li and colleagues found that miR-200c expression was significantly lower in A549, H1299, and SPC-A-1sci non-small cell lung cancer (NSCLC) cells among other NSCLC cells [57]. Transwell invasion assays showed that these cell lines that expressed lower levels of miR-200c had a higher invasive capability than other NSCLC cells. Furthermore when these highly invasive cells were transiently transfected with miR-200c mimics, their invasive abilities were significantly decreased compared to cells transfected with control oligos, which suggests a suppressive role for miR-200c on NSCLC cell invasion. Bioinformatic analysis suggested ubiquitin specific peptidase 25 (USP25) as a potential target for miR-200c and a luciferase reporter assay confirmed the direct binding of miR-200c to its 3′UTR. To further show the importance of this protein in cellular invasion, knockdown of USP25 significantly reduced cell invasion and its overexpression increased cell invasion as assayed by transwell invasion assays.

### Effect of the miR-200 family on epithelial-to-mesenchymal transition and tumor cell migration

One of the most critical properties that a tumor cell must obtain in order to metastasize is the ability to move away from the primary tumor. One of the most widely studied cellular programs that tumors cells activate to gain this motility is known as the epithelial-to-mesenchymal transition (EMT). EMT is a process by which a normally polar, epithelial cell undergoes a change to a mesenchymal-like cell. By undergoing EMT, a cell is able to take on the characteristics of a mesenchymal cell and become more motile and invasive. Accompanying this morphological change is a shift in expressed proteins, and an increase in the production of transcription factors and extracellular matrix degrading enzymes within the cell. EMT occurs naturally during embryogenesis; however it is now thought to be a major contributor to the metastasis of epithelial-originated cancers.

Two of the major hallmarks of EMT include a loss of the epithelial markers such as E-cadherin, a cell-cell adhesion protein, and the increase in the expression of mesenchymal markers such as ZEB1, ZEB2 and other EMT-inducing transcription factors. These two hallmarks are mutually exclusive from the other within an epithelial cell. ZEB1 and ZEB2 act as E-cadherin repressors by directly binding to the E-boxes within the E-cadherin promoter [58–60], thus ZEB1 and ZEB2 are directly involved in the control of EMT by suppressing the expression of E-cadherin, and their expression promotes cell migration and invasion. Recent studies suggest that the miR-200 family is pivotal in regulating EMT by targeting ZEB1 and ZEB2 via direct interactions with their 3′UTRs [61–63]. Through down-regulating ZEB1 and ZEB2 expression, the miR-200 family can effectively up-regulate cellular E-cadherin level and maintain a cell in a more epithelial-like state. However, ZEB1 and ZEB2 can also bind to the E-box sites close to the transcription start site of each of the miR-200 clusters inhibiting their transcription, resulting in a negative feedback loop [64, 65]. Thus, ZEB1 and ZEB2 can keep a cell in a mesenchymal phenotype by repressing the transcription of both E-cadherin and the miR-200 family. Therefore, the interplay between the miR-200 family and ZEB1/ZEB2 plays an important role in driving the cell in to and out of EMT.

Cell migration is a critical step in the metastatic cascade where tumor cells move away from the primary tumor to enter the blood stream. Early studies on the miR-200 family have shown that the miR-200 family can suppress cell migration. Gregory *et al*. first reported the inhibitory effect of miR-200 on cell migration using a transwell migration assay [61]. Using specific miR-200 inhibitors, this group found that the miR-200 inhibitors increased cell migration of Madin-Darby canine kidney epithelial cells, suggesting that the miR-200 family inhibits cell migration. Similarly, Park *et al*. found that expressing miR-200a/c in the highly metastatic MDA-MB-231 breast cancer cells significantly decreased their motility in a transwell migration assay [62]. Both of these studies also showed that cells that have undergone EMT have an increased motility as well as an increase in the expression of the mesenchymal markers ZEB1, ZEB2 and vimentin. Furthermore, these and others have shown that the miR-200 family is a strong inhibitor of EMT, and that EMT resulting from the loss of the miR-200 family depends on ZEB1 and/or ZEB2 up-regulation. Therefore, it was concluded that the miR-200 family elicits this inhibitory effect on cell migration by targeting both ZEB1/2.

However, ours and other recent studies also suggest that the miR-200 family can inhibit cell migration independent of its effect on ZEB1/ZEB2. Li and colleagues found that mammary fat pad injection of the metastatic MDA-MB-231 LM2 breast cancer cells resulted in metastasis to the lung and bone, and this was greatly reduced by the stable expression of miR-200b or miR-200c [43]. This suppressive effect on metastasis was not seen when miR-141 was stably expressed in these cells suggesting that the functional group I miR-200s (miR-200b/-200c/-429) is able to repress metastasis in these cells. To determine whether miR-200 suppresses metastasis by targeting ZEB1, the ZEB1 expressing cDNA lacking the 3′UTR was engineered into the MDA-MB-231 LM2 cells that stably expressed miR-200b. It was found that miR-200b was still able to inhibit metastasis when ZEB1 was forcibly expressed, which implies that miR-200b can inhibit metastasis in a ZEB1-independent manner. Through invasion assays and luciferase reporter assays, it was determined that miR-200b regulates cell migration and metastasis by targeting moesin, and restoration of moesin prevents miR-200b from suppressing cell migration and metastasis.

Our recent studies have also implicated the miR-200 family in the ZEB1-independent regulation of cell migration and metastasis. In the highly migratory arsenic-transformed cells and basal mesenchymal-like triple negative breast cancer cells, we found that PKCα expression levels are significantly higher, and re-expression of miR-200b reduced PKCα levels and inhibited cell migration as well as mammary tumor metastasis [42, 66]. Subsequent luciferase reporter assays revealed that miR-200b directly targets the 3′UTR of PKCα. Moreover, siRNA knockdown of PKCα significantly reduced cell migration. In contrast, enforced expression of PKCα reversed the inhibitory effect of miR-200b on cell migration and tumor metastasis with no significant effect on ZEB1 expression. These findings suggest that miR-200b suppresses cell migration and metastasis by targeting PKCα, which is independent of its effect on ZEB1.

By applying the Ago-HITS-CLIP technology for transcriptome-wide identification of direct miRNA targets in living cells, Bracken *et al* recently identified a good number of miR-200a and miR-200b targets [67]. Further functional validation of the identified miR-200 targets revealed that they constitute subnetworks that play crucial roles in enabling cancer cells to migrate and invade. The identified miR-200 targets are critically involved in Rho-ROCK signaling, invadopodia formation, matrix metalloproteinase activity, and focal adhesions. This work showed for the first time a global regulatory network directly regulated by miR-200 family, which provided a novel mechanistic insight for miR-200 family maintaining the key features of the epithelial phenotype and preventing cell migration.

### Effect of the miR-200 family on tumor cell intravasation

Intravasation is a complex step that involves cancer cells entering the blood vessels or lymphatic system. It can be aided by gene changes that promote the ability of cancer cells to cross the basement and endothelial membranes that form the walls of the vessels. Notch signaling [68] and tumor-associated macrophages (TAMs) [69] are two example mechanisms that have been shown to positively modulate intravasation of cancer cells. In addition to these mechanisms, new blood vessels that have been formed by the primary tumor are leaky and therefore can also facilitate intravasation [70, 71]. It has been shown that stably expressing both miR-200 cluster members reduced the ability of cancer cells to enter the blood stream, and that E-cadherin overexpression can also decrease the number of cells in the blood stream [72]. However, there has been little research done with respect to the mechanism by which miR-200 family reduces cancer cell intravasation. This is probably partly due to the difficulty in measuring intravasated cells in the blood stream, and that the miR-200 family has a strong suppressive effect on the earlier steps of the metastatic cascade. More research is needed to fully understand the intravasation process, and development of a system in which we can directly observe and measure intravasation is critical in doing that.

### Effect of the miR-200 family on tumor cell survival in circulation

After tumor cells have successfully intravasated into the blood stream, they can circulate throughout the body. These tumor cells that have entered the blood stream are known as circulating tumor cells (CTCs). Since the blood stream is such a harsh environment, these CTCs need to adopt a molecular profile that promotes survival in the blood stream. Therefore CTCs often reprogram certain cellular programs such as apoptosis and anoikis to survive. MiR-200 family members have been shown to regulate apoptosis and anoikis, and therefore may have an effect on tumor cell survival in circulation.

In the case of apoptosis, Uhlmann *et al*. found that in MDA-MB-231 breast cancer cells, overexpression of the miR-200b/c/429 cluster significantly reduced cell viability and increased apoptosis [73]. It was further confirmed that PLCγ1 was a direct target of the miR-200b/c/429 cluster, and PLCγ1 knockdown resulted in reduced cell viability and increased caspase activity.

Schickel *et al*. found that stably expressing miR-200c in CAKI-1 kidney and HeyA8 ovarian or transiently expressing it in ACHN kidney cancer cells, caused these cells to be much more sensitive to CD95 (a death receptor)-mediated apoptosis when treated with a CD95 agonist [74]. It was confirmed that miR-200c directly targets FAP-1, a known inhibitor of CD95-mediated apoptosis in these cells, and that the likely mechanism for the sensitivity seen was due to an increase of CD95 surface expression because of FAP-1 suppression. In another study, miR-200c was shown to target Noxa, a member of the Bcl-2 family, in MCF7 breast cancer cells [75]. Through functional studies in these cells it was determined that miR-200c potentiates apoptosis to the clinically used proteasome inhibitor bortezomib by reducing Noxa levels. In addition, in meningiomas it was found that miR-200a directly targeted β-catenin to increase apoptotic cell death [76]. Therefore, downregulation of miR-200 family levels in tumor cells can help the cell survive within the bloodstream by reducing apoptosis.

Anoikis is a kind of apoptosis that is induced by inappropriate or inadequate cell-ECM attachment. Alteration of this complex signaling process can allow tumor cells to survive in the circulatory system that is normally unsuitable for these cells.

Howe *et al*. demonstrated that miR-200c levels were significantly reduced in BT549 and MDA-MB-231 triple negative breast cancer cells [77]. Further work showed that stably expressing miR-200c induces anoikis in these cells by directly targeting TrkB, a neurotrophic tyrosine receptor kinase. Zhang and colleagues also studied the role of miR-200 in anoikis in breast cancer [78]. Transfection of miR-200b in MDA-MB-231, BT549, and Hs578T TNBC cells increased the number of apoptotic cells in suspension-culture. Pin1, peptidylprolyl cis/trans isomerase, NIMA-interacting 1, was confirmed as a direct target of miR-200b, and simultaneously expressing miR-200b and an untargetable Pin1 resulted in a decreased number of cells undergoing anoikis in culture. However, in contrast to these studies, Yu *et al*. found that expression of miR-200a made breast cancer cells more resistant to anoikis [79]. YAP1, yes-associated protein 1, was confirmed to be a direct target of miR-200a and by targeting this protein miR-200a allows cells to avoid anoikis. Given the contradictory results observed, the role of individual members of the miR-200 family in anoikis still needs to be further studied.

By regulating above cellular processes, cancer cells are able to survive in the harsh circulation system. In addition, immune system differentiation, activation, development, or response could also play critical roles in the survival of intravasated tumor cells. However, little research has been done specifically looking at the effect of the miRNA-200s on immune system regulation. Therefore, more research is needed in order to further elucidate the role that these miRNAs play in this critical step of metastasis.

### Effect of the miR-200 family on tumor cell extravasation and metastatic colonization

The final steps of metastasis involve surviving CTCs coming to arrest in the blood vessels, extravasating from the blood vessels, and colonizing a distant organ. In order for extravasation to occur, CTCs must first come in contact the endothelium either by becoming lodged in smaller vessels or by specifically adhering to the endothelium. Once a circulating tumor cell extravasates from the blood vessels (likely facilitated by ZEB1 and N-cadherin expression [80]), these cells have a tendency to undergo mesenchymal-to-epithelial transition (MET) which is most likely due to the absence of the signals they received from the primary tumor to undergo EMT. MET facilitates the settling of a cancer cell at a distant organ by allowing these cancer cells to recover their epithelial properties. Once a cell settles in the distant organ, it begins to proliferate and colonize the organ. In striking contrast to the strong inhibitory effect of miR-200 family has on the early metastatic steps, studies have shown that miR-200 may promote metastatic colonization [72, 81].

To study the role of miR-200 in the last step of the metastatic cascade, Korpal and colleagues profiled the levels of the miR-200 family in primary and metastatic samples and found that the miR-200 family was higher in metastatic secondary tumors [72]. Moreover, profiling a group of mouse breast cancer cell lines (67NR, 168FARN, 4TO7, and 4T1) with different metastatic capabilities (67NR cells are unable to intravasate; 168FARN cells cannot extravasate efficiently; 4TO7 cells do not colonize distant organs well; and 4T1 cells are capable of completing all steps of metastasis) revealed that the most metastatic cells (4T1) had the highest level of miR-200 family expression. Similarly, Dykxhoorn *et al*. reported that the 4TO7 cells, lacking the capability of colonizing distant organs, had almost undetectable expression levels of the miR-200b/c/429 cluster compared to the strongly metastatic 4T1 cells [81], suggesting a potential role for the miR-200b/c/429 cluster in colonization. To study the role of the miR-200s in metastatic colonization, Korpal *et al*. stably expressed cluster I (miR-200b/a/429: which will be referred to as C1), cluster II (miR-200c/141: C2) or clusters I and II (C1+C2) in the weakly metastatic 4TO7 cells. Importantly, it was found when C2 was stably expressed the other cluster (C1) also showed a significant increase in expression levels; however, C1 overexpression did not increase the expression levels of C2 miR-200 members. When 4T1 cells, the parental 4TO7 cells and modified 4TO7 cells were injected into the mammary fat pad of mice, it was found that all mice injected with 4T1 cells developed lung and liver metastases, while no mice injected with parental 4TO7 cells had any detectable metastases [81]. In contrast, injection of the modified C2 or C1+C2 4TO7 cells (cell lines that both express high levels of all five miR-200 members) formed more lung metastases than the parental or C1 alone overexpressing 4TO7 cells [72]. Furthermore, Dykxhoorn *et al*. also found that about 80% of mice injected with C2 4TO7 cells developed lung metastases, indicating that the 4TO7 cells that stably express miR-141/200c act similarly to the metastatic 4T1 cells [81]. In addition, Korpal and colleagues also determined that this increase in metastasis by the stable expression of the miR-200s was not due to the increased E-cadherin expression because E-cadherin overexpression alone in these cells caused no increase in lung metastases that was seen in the C2 and C1+C2 cells [72]. These data suggests that the miR-200 family targets pathways involved in inhibiting metastatic colonization.

To further determine the underlying mechanism behind miR-200 promotion of metastatic colonization, Korpal and colleagues used a tail vein injection model with the modified 4TO7 cells described above. Results from this experiment showed more lung metastases for all cell lines with C2 and C1+C2 cells having the greatest effect. Knockdown of E-cadherin in C1+C2 cells did not affect their metastatic efficiency significantly [72]. This again suggested that other targets of the miR-200 family are more important in colonization efficacy of a cancer cell. Through microarray and mass spectrometry analysis nine potential miR-200 targets were identified. Of these nine targets, three were confirmed as direct targets of miR-200: cofilin 2 (Cfl2), low-density lipoprotein receptor-related protein 1 (Lrp1) and Sec23a, a key component of COPII vesicles. Functional analysis *in vitro* and *in vivo* revealed that miR-200 increased metastatic colonization by targeting Sec23a. Further analysis also revealed that Tinagl14 and Igfbp4, two secreted metastasis suppressors, are directly regulated by Sec23a [72].

Interestingly, a recent study showed that the metastatic 4T1 cells, but not the poorly metastatic 4TO7 cells, can secrete miR-200s into extracellular vesicles (EVs) [82]. Moreover, it was also found that the poorly metastatic 4TO7 cells can take up miR-200 from 4T1 EVs and become metastatic in a miR-200–dependent manner [82]. This study provided novel evidence showing that metastatic capability can be transferred from metastatic to non-metastatic cancer cells through extracellular vesicles. In addition, this finding also suggests that circulating miRNAs are not only just cancer biomarkers; they are also functional being capable of promoting metastasis *in vivo*.

These studies suggest that even though the miR-200 reduces the number of cancer cells in the bloodstream, probably by strongly inhibiting the early metastatic steps, those cancer cells that have high expression levels of miR-200s and do manage to get through the extravasation step are more capable of colonizing a distant organ. Additionally since these studies have shown that it is the overexpression of the miR-200c/141 cluster that causes the increase in metastatic colonization, it is possible that the miR-200c/141 cluster may act as a suppressor for early steps of metastasis, but facilitates post-extravasation events while the miR-200b/a/429 cluster suppresses metastasis at all steps. However, more research is needed to discern the function of each cluster as a whole and to elucidate the effect of each individual member of the miR-200 family on the metastatic cascade.

## The miR-200 family as potential diagnostic and prognostic tools

The search for biomarkers that can serve as an early detection method or as a predictive tool for prognosis is needed to increase the earlier diagnosis and thus the long term survival of cancer patients. The studies of miRNAs as potential diagnostic and prognostic tools mainly stem from reports showing that there are stable, cell-free miRNAs in the blood (termed circulating miRNAs) [83–86], and that circulating miRNAs have specific expression profiles for different cancers, although their origins are currently unclear. Further studies are needed to elucidate whether they come from tumor cell death, are secreted from tumor cells, or originate from blood cells associated with the tumor as well as elucidate their expression patterns in individual cancer types. It is because of this unknown that most studies only show a correlation between a certain cancer and miRNAs.

Studies showed potential in the use of members of the miR-200 family for cancer diagnosis. A genome-wide study by Madhavan *et al*. found that circulating miRNAs can act as diagnostic markers for circulating tumor cells (CTCs) in metastatic breast cancer (MBC) [87]. Their analysis of CTC-positive versus CTC-negative MBC patients revealed distinct miRNA signatures for each group, and consequently 17 of these miRNAs were further studied. Of these 17 miRNAs, four of them were from the miR-200 family (miR-200b, -200a, -200c, and -141), and this group concluded that miR-200b was the best miRNA for determining CTC-positive MBC patients. Similarly, a study by Cheng *et al*. found that plasma miR-141 levels are increased in colorectal cancer, highly associated with stage IV colorectal cancer, and able to increase the detection of Stage IV colon cancer when combined with the commonly used colorectal cancer detection marker carcinoembryonic antigen (CEA) [88]. In contrast, Park *et al*. reported that miR-200a was expressed at lower levels in the saliva of 38 patients with oral squamous cell carcinoma (OSCC) compared to 38 healthy controls [89]. These studies suggest that body fluid miR-200 family levels may have different diagnostic values for different types of cancers.

The use of the miR-200s as a prognostic marker also looks promising. Comparing the expression levels of the miR-200 family members in gastric cancer cell lines, Valladares-Ayerbes and colleagues found that miR-200c levels were much significantly higher in cancer cells compared to normal cells [90]. Further analysis using patient samples showed an inverse correlation between miR-200c blood levels and prognosis, which suggests miR-200c as a potential prognostic biomarker for gastric cancer. Xu and colleagues were able to show that miR-200 family expression was significantly correlated with the status (benign, non-recurrent or recurrent primary, or metastatic) of a melanoma tumor, therefore expanding the potential role of the miR-200 family as a prognostic marker in this disease [91]. In addition, Cheng *et al*. reported that circulating miR-141 levels were negatively associated with overall survival for the colon cancer patients [88]. Furthermore, a high level of circulating miR-141 was found to be associated with high-risk (Gleason score ≥ 8) tumors [92], while a lower level of cluster I of the miR-200 family was correlated with relapse [93] in prostate cancer. High levels of miR-141 were also correlated strongly with decreased overall survival in the luminal subtypes of breast cancer [50]. In contrast, it was found that low expression of cluster I miR-200s (miR-200b, -200a and -429) correlated with poorer overall survival in ovarian and endometrial cancer [94, 95]. Together, these findings suggest that miR-200 levels may have the potential to serve as indicators of cancer prognosis.

It is interesting to note that the majority of data reviewed in previous sections from experimental model systems showing that miR-200s suppress tumor development and progression. However, the clinical diagnostic and prognostic data summarized above are much less consistent about the role of the miR-200s as a suppressor of tumor growth and metastasis. This inconsistence could be due to: (i) the levels of circulating miR-200s, not the levels of miR-200s in cancer tissues in most cases, were used for potential cancer diagnosis and prognosis prediction. However, the origin of circulating miRNA is currently unknown; (ii) it is likely that the role of miR-200s in cancer development and progression may be cancer type-dependent or even cancer subtype-dependent. Indeed, Pecot *et al*. recently reported that higher miR-200 levels in ovarian, lung, renal and basal-like breast adenocarcinomas are associated with improved clinical outcome. However, higher levels of miR-141 are significantly associated with worse clinical outcome of luminal subtypes breast cancer [50], suggesting that miR-200 may exhibit differential functions among different breast cancer subtypes.

## The potential role of miR-200 family in cancer therapy

The idea of miRNAs contributing to chemoresistance has been widely studied [96, 97]. The ability of a cell to avoid apoptosis [98] and to undergo EMT [99] have been shown to contribute to the chemoresistance of tumor cells. With the development of microarrays researchers have been able to determine the expression levels and patterns of miRNAs in chemoresistant cells, and this has allowed researchers to determine potential miRNAs involved in the process of apoptosis and chemoresistance. The role of the miR-200 family in apoptosis has already been discussed above in the survival in circulation section; therefore this section will focus primarily on the effect of miR-200s on chemoresistance.

Chemotherapeutic resistance of cancer cells is thought to be one of the primary causes of recurrence in cancer. Although inadequate delivery of the drug to the tumor can contribute to chemoresistance, cellular reprogramming also plays a major role in establishing this resistance. By turning off genes involved in chemosensitivity and turning on genes involved in chemoresistance, tumor cells can effectively evade the drug. Studies have shown that the miR-200 family plays a role in reducing chemoresistance by targeting these genes known to play a direct role in developing this resistance. Liu *et al*. found that the expression of miR-200c is decreased in melanoma tissues and cells, with a further decrease in metastatic primary melanoma tumors [100]. In depth analysis revealed that miR-200c reduces the expression of ATP-binding cassette (ABC) transporters ABCG2, ABCG5 and MDR1 in WM115A melanoma cells. This is important because of their known involvement in the multidrug resistance seen in cancer [101]. These findings suggest that downregulation of miR-200c may contribute to the development of chemoresistance in melanoma.

By generating a doxorubicin-resistant breast cancer cell line (BT474), Kopp *et al*. also showed that loss of miR-200c is important in developing chemoresistance [102]. It was found that miR-200c was significantly downregulated in doxorubicin-resistant cells. When treated with a miR-200c inhibitor, these doxorubicin-resistant BT474 cells became even more resistant to doxorubicin treatment compared to control cells. In contrast, overexpressing miR-200c resensitized these doxorubicin-resistant breast cancer cells to doxorubicin treatment. Mechanistic studies from this group determined that miR-200c reduces drug resistance in these cells through targeting Neurotrophic Tyrosine Kinase, Receptor, Type 2 (TrkB) and BMI1 Polycomb Ring Finger Oncogene (BMI1) [102]. In addition, another study revealed that loss of miR-200c expression is associated with poorly differentiated endometrial carcinoma; and restoration of miR-200c in a papillary uterine cancer line significantly increased its chemosensitivity to the microtubule-targeting chemotherapeutics paclitaxel, vincristine, and epothilone B [103].

Using a miRNA microarray, Kovalchuk and colleagues found that the levels of miR-200a and miR-200c were significantly lower in MCF-7 breast cancer cells that were resistant to doxorubicin compared to the parental cells, suggesting that decreased expression of these miRNAs may contribute to doxorubicin resistance in breast cancer [104]. Using the same techniques as Kovalchuk *et al*., Pogribny and colleagues found that in MCF-7 cells miR-200b and -200c expression were inversely correlated with resistance to cisplatin [105]. However, in contrast to the findings from above studies, Hamano *et al*. found that miR-200c overexpression induces cisplatin resistance in esophageal cancer cells (TE8-R) [106], suggesting that the relationship between the miR-200c expression levels and chemoresistance may be cellular context and drug dependent.

A miRNA microarray done by Meng *et al*. in cholangiocyte cell lines showed that miR-200b and -141 are dysregulated in malignant cholangiocytes [107]. By culturing cholangiocarcinoma cells (Mz-ChA-1) with gemcitabine in the presence or absence of miR-141 or miR-200b inhibitors, they were able to determine that the inhibition of miR-200b decreased gemcitabine-induced apoptosis. Separately, Rui and colleagues also found that decreased miR-200b levels are associated with resistance to docetaxel in a lung adenocarcinoma cell line (SPC-A1 and SPC-A1/docetaxel) [108]. These findings suggest that decreased expression of miR-200b may play a critical role in chemoresistance.

A recent paper by Manavalan *et al*. has also shown a link between the miR-200 family and targeted therapy resistance in breast cancer cells [109]. The expression levels of the miR-200 family members was determined by qPCR in MCF-7 cells that were either sensitive or resistant (LY2) to endocrine treatment, and showed that the expression of miR-200b, -200a, and -200c was significantly decreased in the endocrine-resistant cell lines. To determine if these miRNAs affected sensitivity to endocrine treatment, the LY2 cells were transiently transfected with their precursor miRNA and cell viability was determined in the presence and absence of the antiestrogens 4-OHT (the active metabolite of tamoxifen) or fulvestrant [109]. Results from this experiment showed that expression of miR-200b and miR-200c enhance the sensitivity of LY2 breast cancer cells to growth inhibition by both 4-OHT and fulvestrant. Therefore, the miR-200 family also plays a role in sensitivity to the specific targeted therapies available for breast cancer.

Though much of the research on the miR-200 family in cancer drug resistance has focused mostly on miR-200b and -200c, it is possible that the other members of the miR-200 family may also play similar roles in the process due to their similar seed sequences.

## Conclusions and perspectives

**Figure 3:**
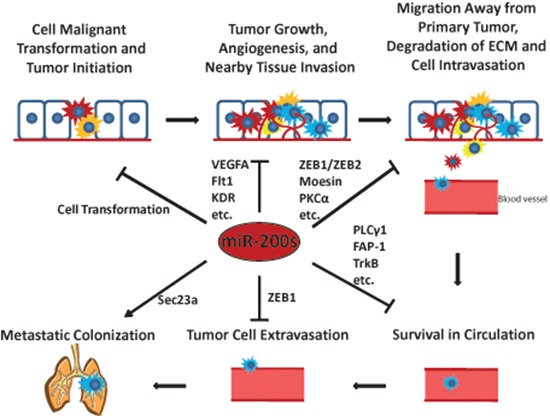
The miR-200s play critical roles in tumor initiation and the metastatic cascade Some representative miR-200 targets involved in each step of the metastatic cascade are shown. ↓ means promote ┴ means inhibit.

Studies on the miR-200 family have enhanced our knowledge of the crucial roles that they may play in cancer development and progression through targeting a variety of important proteins. A comprehensive list of currently validated miR-200 family targets is presented in Table 1. While miR-200s were found to be critically involved in the metastatic colonization to the lungs, current findings in general support the conclusion that the miR-200 family may mainly function as tumor suppressors and metastatic inhibitors as summarized in Figure 3.

**Table 1:** A summary of the validated direct targets of the miR-200 family

Direct targets of the miR-200 family	Cell types	Targeted by which miR-200 family member(s)	Functions of the targets	References
C-Abl Oncogene 2, Non-Receptor Tyrosine Kinase (ABL2)	Breast	miR-200b	Binds F-actin and Microtubules; Plays a Role in Cytoskeletal Rearrangements	[67]
Adaptor-Related Protein Complex 1, Sigma 2 Subunit (AP1S2)	Breast	miR-200a/b	Small Subunit of Clathrin-associated Adaptor Protein Complex 1; Plays a Role in Protein Sorting in Golgi	[67]
Anillin, Actin Binding Protein (ANLN)	Breast	miR-200a/b	Required for Cytokinesis	[67]
B-Cell CLL/Lymphoma 2 (BCL2)	Stomach, Lung	miR-200b/c/429	Anti-Apoptotic Protein; Regulates Cell Death by Controlling Mitochondrial Membrane Permeability	[110]
BMI1 Polycomb Ring Finger Oncogene (BMI1)	Breast, Skin	miR-200b/c	Regulator of Stem Cell Self-Renewal	[44, 111, 112, 113]
Calponin 3, Acidic (CNN3)	Breast	miR-200a/b	Implicated in Regulation and Modulation of Smooth Muscle Contraction	[67]
Catenin (Cadherin-Associated Protein), Beta 1, 88kDa (CTNNB1)	Brain, Liver, Nasopharynx	miR-200a	Involved in the Canonical Wnt Signaling Pathway; Translocates to Nucleus and Interacts with TCF/LEF Family Members to Transcribe Target Genes	[76, 114, 115]
Cell Division Cycle 25B (CDC25B)	Breast	miR-141	Required for Entry into Mitosis	[116]
Chemokine (C-X-C Motif) Ligand 1 (CXCL1)	Ovary	miR-200a/b	Pro-Angiogenic Chemokine For CXCR2; Plays a Role in Inflammation and as a Chemoattractiant for Neutrophils	[50]
Cofilin 2 (CFL2)	Breast, Cervix, Esophagus	miR-200b/a/429	Involved in the Regulation of Actin-Filament Dynamics; Can Bind Both G- and F-actin	[67, 117, 118]
V-Crk Avian Sarcoma Virus CT10 Oncogene Homolog-like (CRKL)	Breast	miR-200b	Activates the Ras and Jun Kinase Signaling Pathways	[67]
Cyclin-Dependent Kinase 2 (CDK2)	Esophagus	miR-200b	Phosphorylates Cancer Related Proteins (CTNNB1, RB1, TP53, MYC)	[117]
Cyclin-Dependent Kinase Inhibitor 1B (CDKN1B)	Colon	miR-200b	Controls Cell Cycle Progression at G1; Modulates Activation of Cyclin A-CDK2, E-CDK2 and D-CDK4 Complexes	[119]
Cyclin-Dependent Kinase Inhibitor 3 (CDKN3)	Breast	miR-200a	Dephosphorylates CDK2; May Play a Role in Cell Cycle Regulation	[67]
Dedicator of Cytokinesis 4 (DOCK4)	Breast	miR-200a/b	Acts as a GEF; Involved in Regulation of Adherens Junctions	[67]
Discoidin, CUB and LCCL Domain Containing 2 (DCBLD2)	Breast	miR-200b	May be Involved in Vascular Remodeling and Influence Vascular Smooth Muscle Cell Proliferation; Plays a Role in Cell Motility	[67]
DNA (Cytosine-5-)- Methyltransferase 3 Alpha (DNMT3A)	Stomach	miR-200b/c	Adds Methyl Groups to DNA; Responsible for CpG Island Methylation; Required to Methylation of Imprinted Loci; Corepresses ZBTB18	[120]
DNA (Cytosine-5-)- Methyltransferase 3 Beta (DNMT3B)	Stomach	miR-200b/c	Adds Methyl Groups to DNA; Responsible for CpG Island Methylation; Activates BAG1; Acts as Corepressor by Associating with ZHX1	[120]
Down-Regulator of Transcription 1, TBP-Binding (Negative Cofactor 2) (DR1)	Breast	miR-200a/b	Can Bind DNA; Acts as a Functional Repressor of Transcription	[67]
E2F Transcription Factor 3 (E2F3)	Kidney, Prostate	miR-200c	Transcription Factor; Activity Inhibited by Rb; When not Bound to Rb it Regulates Genes Involved in the Cell Cycle	[113, 121, 122]
ELK3, ETS-Domain Protein (SRF Accessory Protein 2) (ELK3)	Breast	miR-200a/b	May be a Negative Regulator of Transcription; Can Activate Transcription with Ras, Src, or Mos	[67]
ERBB Receptor Feedback Inhibitor 1 (ERRFI1)	Bladder, Breast	miR-200b/c	Negative Regulator of EGFR Signaling	[67, 123]
C-Ets Avian Erythroblastosis Virus E26 Oncogene Homolog 1 (ETS-1)	Kidney, Lung	miR-200b/c	Transactivator and Transrepressor; Controls the Expression of Cytokine and Chemokine Genes; Implicated in Angiogenesis	[124, 125, 126]
Fas-associated Protein-Tyrosine Phosphatase-1 (FAP-1)	Colon, Ovary	miR-200c	Tyrosine Phosphatase That Negatively Regulates FAS-induced Apoptosis and NGFR-mediated Pro-apoptotic Signaling	[74]
Fermitin Family Member 2 (Kindlin-2) (FERMT2)	Esophagus	miR-200b	Scaffolding Protein that Enhances TLN1/2-mediated Integrin Activation; Required for Focal Adhesion Assembly	[117]
Fibronectin 1 (FN1)	Breast, Endometrium	miR-200c	Involved in Cell Adhesion, Motility, Wound Healing, and Shape by Binding Collagen, Fibrin, Heparin, DNA, and Actin	[127]
Fibulin 5 (FBLN5)	Muscle	miR-200c	Secreted ECM Protein; Promotes Adhesion of Endothelial Cells	[128]
FMS-Related Tyrosine Kinase 1 (FLT1, VEGFR1)	Lung	miR-200b/c	Receptor Tyrosine Kinase; Binds VEGF-A, VEGF-b, and PGF to Regulate Angiogenesis, Cell Survival, Migration, and Invasion	[46, 49]
Formin Homology 2 Domain Containing 1 (FHOD1)	Breast	miR-200c	Required for the Assembly of Stress Fibers; Dependent Upon Rho/ROCK for its Activity	[56]
GATA Binding Protein 2 (GATA2)	Kidney	miR-200b	Zinc-Finger Transcription Factor; Involved in the Development and Proliferation of Hematopoietic and Endocrine Cells	[129]
GATA Binding Protein 4 (GATA4)	Heart, Kidney, Muscle	miR-200b/c	Zinc-Finger Transcription Factor; Involved in Embryogenesis and in Myocardial Differentiation and Function	[130, 131]
Guanine Nucleotide Bind Protein, Alpha Inhibiting Activity Polypeptide (GNAI3)	Breast	miR-200a/b	Reduces Adenylyl Cyclase Activity	[67]
Hermansky-Pudlak Syndrome 5 (HPS5)	Breast	miR-200a/b	May Regulate Synthesis and Function of Lysosomes and Highly Specialized Organelles; Regulates Intracellular Trafficking in Fibroblasts	[67]
Inhibitor of Kappa Light Polypeptide Gene Enhancer in B-cells, Kinase β (IKKβ, IκBKB)	Endometrium, Muscle	miR-200c	Activates NFκB by Phospohorylating its Inhibitor (IκB)	[132, 133]
Interleukin 8 (IL8)	Ovary	miR-200a/b	Potent Angiogenic/Chemotactic Factor	[50]
Jagged 1 (JAG1)	Breast, Pancreas	miR-200c/141	Ligand for Notch 1; Involved in Hematopoiesis	[134]
JAZF Zinc Finger 1 (JAZF1)	Breast	miR-200b	Transcriptional Repressor	[67]
C-Jun Proto-Oncogene (JUN)	Breast	miR-200b	Complexes with c-Fos to Form the AP-1 Transcription Factor	[67]
Kelch-Like ECH-Associated Protein 1 (KEAP1)	Breast	miR-200a	Part of E3 Ligase Complex Involved in Nrf2 Degradation	[135]
Kinase Insert Domain Receptor (KDR, VEGFR2)	Kidney, Lung	miR-200b/c	Receptor Tyrosine Kinase; Binds VEGF-A, VEGF-C, and VEGF-D to Regulate Angiogenesis, Vascular Development, and Embryonic Hematopoiesis	[46, 48, 125, 129]
Kruppel-Like Factor 9 (KLF9)	Endometrium	miR-200c	Transcription Factor; Binds GC Elements in Promoters; Activates when Bound Tandem Repeated GC; Inhibits When Bound Only One GC Box	[132]
Leptin Receptor (LEPR)	Breast, Endometrium	miR-200c	Stimulates Gene Transcription Through STATs; Involved in Fat Metabolism and Hematopoiesis	[127]
Leucine-rich Repeat Containing G Protein-coupled Receptor 4 (LGR4)	Breast	miR-200a	Receptor for R-spondins that are Involved in Wnt Signaling	[67]
Lipoma HMGIC Fusion Partner (LHFP)	Breast	miR-200b	Fused to the High-mobility Group Gene HMGA2 in Many Lipomas	[67]
Low-Density Lipoprotein Receptor-Related Protein 1 (LRP1)	Breast, Cervix	miR-200b/a/429	Involved in Intracellular Signaling, Lipid Homeostasis, and Clearance of Apoptotic Cells via Endocytosis and Phagocytosis	[118]
Mastermind-Like 2 (MAML2)	Breast, Pancreas	miR-200c/141	Transcriptional Coactivator for Notch Proteins; Amplifies Notch-induced Transcription of HES1	[134]
Mastermind-Like 3 (MAML3)	Breast, Pancreas	miR-200c/141	Transcriptional Coactivator for Notch Proteins; Amplifies Notch-induced Transcription of HES1	[134]
Matrin 3 (MATR3)	Adrenal Gland	miR-200b	Nuclear Matrix Protein; Interacts With Other Nuclear Proteins and Regulates Transcription	[136]
Mitogen-Activated Protein Kinase 14 (MAPK14, p38α)	Ovary	miR-200a/141	Mitogen Activated Kinase; Involved in the Regulation of Processes such as the Cell Cycle, Reponse to Stress, Protein Turnover, and Endocytosis	[137]
Mitogen-activated Protein Kinase Kinase 4 (MAP2K4)	Breast	miR-200a	Required for Maintaining Peripheral Lymphoid Homeostasis; Phosphorylates Downstream MAPK Targets	[67]
Mitogen-activated Protein Kinase Kinase Kinase 1 (MEKK1)	Breast	miR-200b	Mediates Cold, Salt, Cadmium and Wound Stress Signaling; Phosphorylates MEK1	[67]
Mitogen-activated Protein Kinase Kinase Kinase 7 (MAP3K7)	Breast	miR-200a/b	Controls a Variety of Cell Functions Including Transcription and Apoptosis; Involved in Response to Environmental Stresses	[67]
Moesin (MSN)	Breast, Endometrium	miR-200b/c	Regulates Actin Localization and Cross-Linking to the Plasma Membrane; Localized to Filopodia	[67, 43, 127]
Myoferlin (MYOF)	Breast	miR-200b	Associates with both Plasma and Nuclear Membranes; Involved in Calcium-mediated Membrane Fusion Events	[67]
Myosin Light Chain Kinase (MYLK)	Breast	miR-200b	Implicated in Smooth Muscle Contraction; Phosphorylates Myosin Light Chains; Involved in the Inflammatory Response, Cell Motility, and Cell Morphology	[67]
Myosin Phosphatase Rho Interacting Protein (MPRIP)	Breast	miR-200b	Targets Myosin Phosphatase to the Actin Cytoskeleton; Required for RhoA and ROCK1 Actin Cytoskeleton Regulation	[67]
Myosin Phosphatase Target Subunit 1 (MYPT1)	Breast	miR-200a/b	A Regulatory Subunit of Myosin Phosphatase 1	[67]
Neuroepithelial Cell Transforming 1 (NET1)	Breast	miR-200b	Guanine Nucleotide Exchange Factor (GEF) for RhoA; May be Involved in Activation of SAPK/JNK pathway	[67]
Neurotrophic Tyrosine Kinase, Receptor, Type 2 (Trkβ) (NTRK2)	Breast, Endometrium	miR-200c	RTK Involved in the Regulation of Differentiation, Proliferation, Survival, Learning, Memory, and Apoptotic Signaling Cascades	[77, 127]
Neurotrophin 3 (NTF3)	Breast	miR-200c	Controls Survival and Differentiation of Neurons; TrkB Ligand	[77, 127]
Notch 1 (NOTCH1)	Breast	miR-200b	Involved in Controlling Cell Fate	[67]
One Cut Homeobox 2 (ONECUT2)	Colon	miR-429	Transcriptional Activator; Involved in Melanocyte and Hepatocyte Differentiation; Activates the Transcription of Liver Specific Genes	[40]
PCNA-Associated Factor 15 (PAF, KIAA0101)	Esophagus	miR-200b	PCNA-Binding Protein that Acts as a Regulator of DNA Repair During Replication	[117]
Peptidylprolyl Cis/Trans Isomerase, NIMA-Interacting 1 (PIN1)	Breast	miR-200b/c	Regulates Mitosis; Catalyzes pSer/Thr-Pro Cis/Trans Isomerizations; Down-regulates Kinase Activity of BTK; Required for RAF1 Dephosphorylation	[78, 138]
Peroxiredoxin 2 (PRDX2)	Lung	miR-200c	Enzyme that Reduces Peroxide and Alkyl Hydroperoxides; Stabilizes Hemoglobin	[139]
Phorbol-12-Myristate-13-Acetate-Induced Protein 1 (PMAIP1, NOXA)	Breast	miR-200c	Promotes Activation of Caspases and Apoptosis; Contributes to p53-dependent Apoptosis After Radiation; Promotes MCL1 Degradation	[75]
Phosphatase and Tensin Homolog (PTEN)	Endometrium, Pituitary Gland	miR-200a/c	Tumor Suppressor; Dephosphorylates Tyrosine-, Serine-, and Threonine-Phosphorylated Proteins; Antagonizes PI3K-Akt/PKB Signaling Pathway	[140, 141]
Phospholipase C, gamma 1 (PLCG1)	Breast	miR-200b/c/429	Forms IP3 and DAG; Involved in Proliferation, Apoptosis, and EGFR-driven Invasion	[67, 73]
Polycystic Kidney Disease 1 (PKD1)	Kidney	miR-200a/b	Regulator of Calcium Permeable Cation Channels and Intracellular Calcium Homeostasis; Involved in Cell-Cell and Cell-Matrix Interactions	[142]
Potassium Channel Tetramerization Domain Containing 2 (KCTD2)	Breast	miR-200a/b	Thought to be Involved in Transcriptional Regulation via Control of Chromatin Structure and Function	[67]
Prominin 1 (CD133)	Brain	miR-200b	Binds Cholesterol; May Play a Role in Apical Plasma Membrane Organization of Epithelial Cells; Regulates MAPK and Akt Signaling	[143]
Protein Kinase C alpha (PRKCA)	Breast, Lung	miR-200b	Serine- and Threonine-Specific Kinase; Shown to Play a Role in Cell Adhesion, Transformation, Migration, and the Cell Cycle Checkpoint	[42, 66]
Protein Phosphatase Mg2+/Mn2+ dependent, 1F (PPM1F)	Breast	miR-200c	Dephosphorylates and Deactivates CaM-kinase II; Mediates Caspase-Dependent Apoptosis	[56]
RAB18, Member RAS Oncogene Family (RAB18)	Breast	miR-200b	Involved in Endocytosis and Recycling	[144]
RAB21, Member RAS Oncogene Family (RAB21)	Breast	miR-200b	Involved in the Control of Integrin Trafficking	[144]
RAB23, Member RAS Oncogene Family (RAB23)	Breast	miR-200b	Antagonist of Sonic Hedgehog Signaling	[144]
RAB3B, Member RAS Oncogene Family (RAB3B)	Breast	miR-200b	Promotes Cancer Cell Survival	[144]
Rho-Associated, Coiled-Coil Containing Protein Kinase 2 (ROCK2)	Breast, Liver	miR-200b/c	Kinase Activated by Rho GTPases; Regulates Actin Cytoskeleton Organization and Cell Polarity	[67, 145]
Rho Family GTPase 3 (RND3)	Colon	miR-200b	Little Known; Binds GTP, but Lacks GTPase Activity; May Act as Negative Regulator of Cytoskeletal Organization	[119]
Rho GTPase Activating Protein 19 (ARHGAP19)	Breast, Endometrium	miR-200c	Function Relatively Unknown; GTPase Activator for the Rho-GTPases	[127]
Rho GTPase Activating Protein 29 (ARHGAP29)	Breast	miR-200b	GTPase Activator for the Rho-GTPases; Strong Activity Towards RhoA; Weak Activity Towards Rac1 and Cdc42	[67]
Rho Guanine Nucleotide Exchange Factor (GEF) 3 (ARHGEF3)	Breast	miR-200b	Acts as a Guanine Nucleotide Exchange Factor (GEF) for RhoA and RhoB GTPases	[67]
Ring Finger Protein 38 (RNF38)	Breast	miR-200a	Acts as an E3 Ubiquitin-protein Ligase	[67]
Sec23 Homolog A (SEC23A)	Breast, Cervix	miR-200b/a/429	Metastasis Suppressor; Component of COPII Vesicles; Involved in Anterograde Transport	[67, 72]
SHC SH2-Domain Binding Protein 1 (SHCBP1)	Breast	miR-200b	May Play a Role in Cell Proliferation, Growth, and Differentiation; Acts as a Positive Regulator of FGF Signaling in Neural Progenitor Cells	[67]
Single-Minded Family BHLH Transcription Factor 2, Short Isoform (SIM2-s)	Brain	miR-200a	Transcription Factor That May be a Master Gene of CNS Development	[146]
Signal Transducer and Activator of Transcription 5b (STAT5B)	Kidney	miR-200a	Acts as a Transcriptional Activator once Phosphorylated; Involved in TCR Signaling, Apoptosis, Adult Mammary Gland Development, and Sexual Dimorphism of Liver Gene Expression	[147]
SRY (Sec Determining Region Y)-Box 2 (SOX2)	Colon, Kidney	miR-200c	Transcription Factor; Involved in the Regulation of Embryonic Development and in Determination of Cell Fate; Required for Stem Cell Maintanence	[121, 148]
SUZ12 Polycomb Repressive Complex 2 Subunit (SUZ12)	Breast, Liver	miR-200b/c	Polycomb Group Protein Involved in Silencing Gene Targets by Methylation of Histone H3	[44, 145, 149]
Tissue Inhibitor of Metalloproteinase 2 (TIMP2)	Muscle	miR-200c	Inhibitor of Matrix Metalloproteinases; Suppresses Proliferation of Endothelial Cells	[128]
Transcription Factor 12 (TCF12)	Breast	miR-200b	Transcriptional Regulator; Involved in Neuronal Differentiation	[67]
Transforming Growth Factor β2 (TGFβ2)	Colon	miR-141	Suppressor of IL-2 Dependent T-cell Growth; Activator of EMT	[65]
Transmembrane Protein 209 (TMEM209)	Breast	miR-200a/b	Nuclear Envelope Protein; Plays a Role in Promotion of Cell Proliferation	[67]
Dynamin Binding Protein (DNMBP, TUBA)	Breast	miR-200a/b	Links Dynamin with Actin-regulating Proteins; Plays a Role in Regulation of Cell Junctions	[67]
Tubulin, β 3 class III (TUBB3)	Endometrium, Ovary	miR-200c	Major Constituent of Microtubules; Plays a Role in Proper Axon Guidance and Maintenance	[150, 151, 152]
Vascular Endothelial Growth Factor A (VEGFA)	Endometrium, Lung, Muscle	miR-200b	Growth Factor that Induces Angiogenesis, Vasculogenesis, and Endothelial Cell Growth; Promotes Migration; Inhibits Apoptosis	[46, 128, 132]
WAS Protein Family, Member 3 (WAVE3)	Breast, Colon, Prostate	miR-200b	Protein Involved in Actin Cytoskeleton Remodeling; Required for the Control of Cell Shape	[55]
WAS/WASL Interacting Protein Family, Member 1 (WIPF1)	Breast	miR-200b	Important in Organization of the Actin Cytoskeleton; Plays a Role in the Formation of Cell Ruffles	[67]
Wingless-Type MMTV Integration Site Family, Member 1 (WNT1)	Stomach	miR-200b	Function Relatively Unknown; Ligand for the Frizzled Receptor Family; Probable Role in Developmental Processes	[153]
X-Linked Inhibitor of Apoptosis (XIAP)	Stomach, Lung	miR-200b/c/429	Potent Apoptosis Suppressor; Directly Binds and Inhibits Caspase-3, -7, and -9 Activity	[110]
Yes-Associated Protein 1 (YAP1)	Breast	miR-200a	Transcriptional Coactivator and Corepressor; Effector of Hippo Signaling Pathway; Regulates Cell Proliferation, Death, and Migration	[79]
Zinc Finger E-box Binding Homeobox 1 (ZEB1)	Breast, Colon, Cervix, Ovary	All five members	Transcriptional Repressor of E-cadherin and miR-200 Family Members; Expression Promotes EMT	[67, 61, 62, 72]
Zinc Finger E-box Binding Homeobox 2 (ZEB2, SIP1)	Breast, Colon, Cervix, Ovary	All five members	Transcriptional Repressor of E-cadherin and miR-200 Family Members; Expression Promotes EMT	[61, 62, 72]

Note: Only those that were confirmed to be a direct target by a luciferase reporter assay in a cell line were presented here. Targets are arranged alphabetically. Gene name is shown followed by gene abbreviation in parentheses. Cell type is the tissue origin of the cell that the luciferase reporter was performed in. Known function is the general known function of the gene.

Although current studies on the miR-200 family have shown promising results, more work is needed to further understand the role this family plays in cancer. Future work on the miR-200 family can help with better understanding the mechanism by which miR-200s affect cancer initiation, metastasis, and relapse. Since much work has focused on the effect of whole clusters/groups on metastasis, more work is also needed to be done on individual members of the miR-200 family to elucidate their role in each step of the metastatic cascade. Developing new and robust models for the study of intravasation and extravasation steps of metastasis are also needed to advance our knowledge on the miR-200′s role in these critical processes. Moreover, most of the research done on individual miR-200 family members focuses on miR-200b or -200c, therefore more work is needed on miR-200a, -141 and -429 and their individual role in cancer. Since some of the data on the role of miR-200 family in cancer is controversial and cellular context dependent, it is important for future studies to tease apart which miR-200 family members act as a tumor suppressor and which may promote cancer progression. Completing these studies will lead to the discovery of more miR-200 targets and ultimately the development of novel and targeted therapeutic options for the treatment of cancer.

## Abbreviations

3′UTR: 3′ untranslated region
4-OHT: 4-Hydroxytamoxifen
BPDE: Benzo(*a*) pyrene diolepoxide
CEA: Carcinoembryonic antigen
CTCs: circulating tumor cells
DGCR8: DiGeorge syndrome critical region 8
ECM: Extracellular matrix
EMT: Epithelial-to-mesenchymal transition
HBECs: Human bronchial epithelial cells
HUVECs: Human umbilical vein endothelial cells
MBC: Metastatic breast cancer
MET: Mesenchymal-to-epithelial transition
miRNA: microRNA
mRNA: messenger RNA
NMU: *N*-Nitroso-*N*-methylurea
NSCLC: Non-small cell lung cancer
OSCC: Oral squamous cell carcinoma
pre-miRNA: precursor miRNA
pri-miRNA: primary microRNA
qPCR: Quantitative polymerase chain reaction
RISC: RNA-induced silencing complex
SCID: Severe combined immunodeficiency
TAMs: Tumor associated macrophages
TNBC: Triple negative breast cancer
Zinc finger E-box binding homeobox 1 and 2: ZEB1 and ZEB2
